# Another pandemic disaster looms: yellow fever spreading from Angola

**DOI:** 10.11604/pamj.2016.24.107.9921

**Published:** 2016-05-31

**Authors:** John Payne Woodall

**Affiliations:** 1 ProMED-mail, International Society for Infectious Diseases, Brookline MA, USA

**Keywords:** Yellow fever, pandemic, Angola, Aedes

## Editorial

On 23 May 2016 Dr Margaret Chan, Director-General of the World Health Organization, declared before the World Health Assembly in Geneva:

“For more than a decade, WHO has been warning that changes in demography and land use patterns in Africa have created ideal conditions for explosive outbreaks of urban yellow fever (YF). Africa's urbanization has been rapid and rampant, showing the fastest growth rates anywhere in the world” [1].

And for more than a decade, some of us in public health have been concerned about the increasing threat of the introduction of YF into Asia. But during all that time, market forces have kept the supply of YF vaccine below global demand.

In Angola, migrant workers from rural mining and construction sites can carry the disease into urban settings, where “powder-keg conditions” exist: dense populations of non-immune residents, heavy infestations with mosquitoes that are suited to urban life, and weak infrastructure that makes mosquito control almost impossible, Chan added.

By the time this appears in print the world will probably be in the grip of a pandemic that will claim many more lives than Ebola, because instead of being spread by contact like Ebola, it is spread by the bites of Aedes mosquitoes, the same ones that are spreading dengue unchecked throughout the tropical and semi-tropical regions of the world.

Under WHO's revised International Health Regulations, yellow fever (YF) and other infectious diseases of international concern should be reported to WHO as soon as a case is suspected, so that WHO can mobilize expert advice and assistance. Confirmation should be obtained as soon as possible afterwards. But when a suspect case of YF appeared in Luanda, the capital of Angola, there were delays in reporting and taking preventive measures, just like what happened with Ebola -- and now YF is spiraling out of control. It has crossed the border into the Democratic Republic of the Congo as far as the capital, Kinshasa, reportedly causing 44 laboratory-confirmed cases (42 in Angolans) as of 19 May, and one autochthonous case in the capital, Kinshasa. It has also reached as far as Mauritania and Kenya in arriving Angolans, fortunately without spread in those two countries as of late May [2]. But the rest of urban Africa, wherever urban Aedes are found, is under threat

### Vaccine

Unlike the Ebola situation, there is a vaccine, but although WHO‘s entire stockpile of around 7 million doses was sent to Angola and mass vaccination began in Luanda in early February, there were thousands of suspect cases and hundreds of deaths in Luanda, and full coverage of the capital had still not been achieved by 19 May. It has now spread to almost all of Angola‘s 18 provinces, with official figures of 2420 cases (736 confirmed) with 298 deaths. [2]. Angola‘s population is about 24 million, and there is not enough vaccine available from manufacturers‘ stocks now, or that can be produced in the coming months with current capacity. Increasing production rapidly is problematic, since it can take up to six months to produce a batch of usable vaccine. Yellow fever vaccines can be stored for up to three years, but manufacturers can′t afford to over produce [3].

The Angolan Health Minister, Luís Gomes Sambo, reported to the World Health Assembly in Geneva (Switzerland), on 23 May 2016 the efforts of the Angolan Government and partners in the fight against the yellow fever outbreak. “A total of 298 deaths were reported, the age group between 15 and 29 years the most affected”, he said, explaining that the *Aedes aegypti* ital mosquito exists almost countrywide. The minister went on to say that the government had developed a National Response Plan which includes five main components: surveillance of the disease, vaccination, integrated vector control, clinical care and social mobilization. He announced that the Government, which considers the outbreak as a priority, had provided approximately USD 70 million to cover the cost of yellow fever vaccines, essential drugs and operating expenses, and informed the Assembly that the Angolan government has decided to vaccinate the entire population. About eight million people have already been vaccinated; recording at this time a decreasing trend in the number of new cases and deaths [4].

Not mentioned were the facts that the figures for cases and deaths are probably underestimates by a factor of 10, according to WHO‘s own expert after a visit in March, so that two months ago there were already thousands of cases and many hundreds of deaths [5]. Nor that to cover the whole population another 16 million doses of vaccine are needed.

WHO says it that it shipped 11.7 million doses to Angola on 18 May, and could have 17 million doses available by August. However, Namibia has requested 450,000 doses and Zambia 50,000 for prevention, and Bolivia, Brazil and Peru are asking for vaccine for sylvan yellow fever outbreaks (not connected with Angola) in those countries – so where will it all come from? Congo DR and Uganda belong to the GAVI Alliance and are expected to receive their vaccine requirements through GAVI, but it is not clear how many doses will be available to them.

The first cases were seen on 5 December 2015 but reported in the Angolan press only on 20 January 2016 and posted on the internet two days later by ProMED-mail [6]. Angola notified WHO on 25 January, and WHO reported it on Disease Outbreak News on 12 February. So it was weeks before the outbreak was officially declared. Three months later, as of 19 May, vaccination of Luanda province‘s population had still not been completed. The figure of 125% coverage for Viana, where the index cases were detected, shown on the map in WHO‘s YF situation report of 26 May, is probably inflated by people flocking to the capital from elsewhere to get protection or travel out of the country [2].

Vaccination has been started in other Angolan provinces, but the Minister of the Interior stated vaccine provided free or at half price to the government is being sold for profit on the black market, where people who can afford to pay can circumvent the long queues at hospitals. Doctors and nurses are implicated, and some of the vendors have been arrested. It is very likely that stolen vaccine has not been stored at the correct temperature to ensure its potency, so it will be ineffective, and that it has been injected using a recycled syringe, risking transmission of hepatitis and HIV/AIDS, so that people who received stolen vaccine are not protected against YF.

YF undergoes cyclical re-emergence in its endemic habitat, where its reservoir is in monkeys and forest mosquitoes. In April an outbreak unrelated to that in Angola appeared in Uganda on the shore of Lake Victoria, unexpectedly far from the eastern edge of the equatorial forest. Sequencing has shown that the strain is of local origin, but it may portend more African outbreaks even beyond the YF endemic zone, with a need for yet more vaccine.

Three YF-infected Angolan travelers were reported on to have arrived in Kinshasa, Congo DR, with spread to one resident by 11 April (probably the tip of an iceberg), and both the government and WHO are warning that the epidemic could now spread there. It is hoped that the delay between notification and vaccination will not be as long as it was in Angola, with its disastrous results.

Other African countries currently considered as high risk yellow fever areas are; Benin, Burkina Faso, Burundi, Cameroon, Central African Republic, Chad, Congo Republic, Ivory Coast, Equatorial Guinea, Ethiopia, Gabon, Gambia, Ghana, Guinea, Guinea-Bissau, Kenya, Liberia, Mali, Mauritania, Niger, Nigeria, Senegal, Sierra Leone, Southern Sudan, Sudan, Togo and Uganda. Namibia and Zambia are requesting vaccine for prevention. But there will still be nowhere near enough. Existing coverage with YF vaccine is low in those few high risk countries which do vaccinate; Nigeria has only 50% coverage of its population, Angola only 18% coverage of its eligible 1-5-year-old children in 2014. Two independent studies published in 2013 showed that vaccine supplies, which are manufactured deliberately over-strength, can be expanded at least five-fold simply by giving a one-fifth dose, which affords full protection to adults [1]. This could be authorized as an emergency measure by the WHO Emergency Use Assessment and Listing procedure (EUAL). The manufacturers are trying hard to increase production -- and vaccination production could be started or revived, for example in India, which used to produce it, if work is begun now -- but it all takes time, and every day that passes without vaccination means YF will spread to more areas and more people will die.

We understand that WHO will consider the question of a five-fold expansion in June, during which another month‘s supply of vaccine that could have been expanded five times will be irrevocably used up. But surely the whole point of emergency measures is to immediately make exceptions to the routine approval process?

### Mosquito control

Other emergency measures that could be implemented are mosquito control. Small fish that eat mosquito larvae are useful in cisterns and rainwater barrels, but not in small breeding sites such as plant axils, flower pots, animal watering containers, tires and trash left out in the rain. Use of transgenic mosquitoes, mosquito pathogens such as *Bacillus thuringensis, Wohlbachia* bacteria and hormones to attack the larvae of the urban mosquito will take too long to scale up and apply. Fogging with chemical pesticides provides temporary relief but does not affect eggs waiting to hatch and produce the next generation. There is a special oil for that. Mycoinsecticides are of much lower toxicity than chemicals. However, the persistent pesticide DDT is still legal for use in India, and WHO has the possibility of approving its use around breeding sites (peri-focal spraying), which should urgently be authorized for use in addition to vaccine. Suspend PolyZone, a long-lasting formulation of deltamethrin (not as persistent as DDT) is also available. It features a proprietary polymer layer that protects the active ingredient from weather, irrigation and mechanical abrasion. This controlled release formulation, which resists erosion, ensures treatment will continue to control targeted pests for up to 90 days outdoors. It also stays right where it is sprayed.

DRC is asking people to use bed-nets, but as the YF mosquito is a day-biter, LLINs (long-lasting insecticidal nets) are only useful for preventing spread from patients. However, they also prevent malaria, transmitted by night-biting Anopheles, and the rains – bringing the malaria season - have now started. Severe cases of malaria can be confused with YF, and lead to misdiagnosis in the absence of blood tests.

Implementing vector control measures will be a challenge, but massive community participation in collection and disposal of trash should be relatively low-cost, and should be started right away in at-risk countries. That would also have the beneficial side effect of reducing the burden of dengue, chikungunya and Zika on overstretched health services, both public and private,

A serious problem is the loss of revenue from oil and gas, which has led to Angola cutting back on collection of street litter in which mosquitoes breed, and the global downturn in economies is affecting all African countries and their health and sanitation budgets.

### Threat to Asia

On top of Africa‘s immediate problems, an even worse scenario is unfolding in China. At least 11 cases of YF in Chinese construction workers and business people returning from Angola have arrived home for treatment, the first time in history that YF has been confirmed in Asia [7]. China has around 50 million people at risk in its dengue-endemic south once summer arrives; one province had 40,000 cases of dengue in a recent year [8]. If YF spreads to the megacities in the tropical dengue belt of Asia, where the YF mosquito is active year-round, it will be a tremendous disaster.

International donors should remember that a dollar spent on prevention at the source saves many times that amount in prevention and treatment at the donor‘s home. This is, of course, provided the funds reach their intended target. Audits of international aid have shown problems with that elsewhere in Africa [9].

Desperate situations demand desperate remedies. Vaccination coverage of 80% is needed to stop an epidemic. Let us hope use of a five-fold lower dose of vaccine can be implemented in time to stall this grave threat.
